# Honours and the Nursing Profession

**Published:** 1916-01-22

**Authors:** 


### The Hospital

The Workers' Newspaper of Administrative Medicine and Institutional Life, Administration, National Insurance and Health.

No. 1545, Vol. LIX. SATURDAY, JANUARY 22, 1916. PRICE ONE PENNY.

### HONOURS AND THE NURSING PROFESSION.

Recent *Gazettes* have been of deep interest to members of the nursing profession. Practically for the first time matrons, sisters, and nurses who have won their way as workers in civil hospitals have at length obtained recognition, not only for distinguished service in the field, but for valuable service in connection with the war. The names recorded in the List of Honours include those of some of the most distinguished and able women who have for years rendered yeoman service to the sick in the voluntary hospitals of this country. The Honours Lists exhibit one grave defect, and that is the omission of the name of the only Principal Matron representative of the Poor-Law infirmaries and nursing service. This lady is one of the ablest, most retiring, and hard-working of women.

The Government and the country owe very much indeed to the whole-hearted way in which the authorities responsible for the Poor-Law infirmaries have suffered every kind of inconvenience and difficulty in order adequately and speedily to meet the demand for an ever-increasing number of hospital beds for the reception of the wounded. Further, the medical superintendents, the matrons, and the sisters, with the nursing staffs of Poor-Law infirmaries, have cheerfully added immensely to their work, and have unsparingly laboured night and day to make the provision for our wounded warriors in these institutions of the very best. No doubt the list of recipients of the Royal Red Cross (First Class) is a long one, but it has lost in authority and dignity by the omission of the name of Miss Eleanor Barton, the Principal Matron of the 3rd London General Hospital. Not only is she the representative of the Poor-Law nursing service, but she is one of the most efficient and competent matrons of the day. On public grounds alone not a moment's delay should be suffered before this grave matter is adjusted by an act of simple justice and the wisest policy.

It is our privilege to offer sincere congratulations to the matrons and others whose work we have known and tested continuously for years. The List of Honours as it stands, when judged by merits and service, is indeed a brilliant one, full of encouragement and pride for every worker throughout the nursing field. It would be invidious to individualise, but we may enforce the truth of this contention by adding our congratulations to the authorities of the following voluntary hospitals whose matrons have been honoured:—King's College, Royal Free, Birmingham General, Bristol Royal Infirmary, Addenbrooke's Cambridge, King Edward VII. Hospital, Cardiff, Croydon General, Leeds General Infirmary, Manchester Royal Infirmary, Newcastle Royal Victoria Infirmary, Oxford Radcliffe Infirmary, Sheffield Royal Infirmary, Aberdeen Royal Infirmary, the Glasgow Royal and Western Infirmaries. This list is of the highest in the administrative sense, and includes an excellent record of most efficiently administered provincial and Scottish hospitals. We may state with absolute conviction that the selection of recipients for honours is one of the most difficult tasks for anyone to fulfil. This knowledge, and the facts just stated, demonstrate the inestimable value to the people of the considerable technical and other knowledge of hospital work and workers, and the deep practical interest in their efficiency, which inspires His Majesty King George V.

It was impossible, of course, to include in the present Honours List every worker whose services merit distinction. There is no type of hospital which has rendered greater or more zealously efficient service to our sailors and soldiers than the voluntary hospitals of this country. These great civil hospitals have yet to be honoured through their non-territorial matrons and the members of their nursing staffs, who have put into the work an extraordinary amount of personal service and self-denial in providing for the needs of our warriors. The matrons and lady superintendents have put themselves out and added greatly to their continuous worries and work by providing a plan whereby short-time probationers and V.A.D. workers have been prepared to render service in the military and other hospitals at home and abroad. The authorities of the voluntary hospitals have incurred grave responsibility and faced heavy expenditure to enable them to offer the best and most perfectly equipped hospital accommodation and hospital service for the succour of the wounded. St. Thomas's Hospital, with its special temporary accommodation for 500 military patients, is one instance of the splendid service rendered by the voluntary hospitals. The extended and various services rendered by St. Bartholomew's Hospital is another example, whilst Guy's Hospital has prepared special accommodation for wounded officers, and given material aid by making rejected recruits efficient for service. These three examples are but types of the splendid work which the voluntary hospitals throughout the United Kingdom have rendered in the most patriotic and courageous manner. To mention these things is but to indicate that the many matrons and other workers in voluntary hospitals have rendered and are rendering invaluable service in connection with the war. At the proper time they are likely to receive the recognition they deserve for their distinguished and devoted labours for our sick and wounded warriors.

